# Event and Comment

**Published:** 1906-08-15

**Authors:** 


THE
### DENTAL REGISTER.

Vol. LX. August 15, 1906. No. 8.

### EVENT AND COMMENT.

### Death from Gum Infection.

The *British Dental Record*, for July, contains an account of an inquest to determine the cause for the death of a man who died three weeks after having his teeth extracted, from what the attending physician called gangrene of the mouth. It seems that the patient had several abscessed teeth and he called upon one of the so-called dental companies to have them extracted. A young man made the operation who was not a registered dentist; in fact, he had simply worked at the business for two and one-half years, and had never been "articled" as an apprentice. His statement is that he injected the gums with a solution prepared by the firm, which contained gaultheria, menthol, phenol and thymol. He stated that the patient suffered no pain during the extraction of twenty or more teeth, as the teeth were easily extracted. The patient, however, according to his wife's statement, was drowsy all the time after the operation for nearly two weeks and his mouth was very sore and painful. A physician was called and treated the patient until his death on a diagnosis of blood poisoning. The physician claimed that the cause of death was due to blood poisoning from gangrene in the jaws, caused by the extraction of too many abscessed teeth at one time; and the verdict of the coroners jury was substantially the same.

The English journals give much of their space to recording accidents of similar character and to the prosecution of illegal practitioners, and it seems strange to us that they will follow up a graduate of an American dental college, who is qualified to practice the art of dentistry intelligently, with such extreme persistence as to prohibit him from practicing in their country and yet they allow such tyros to mutilate their people, who do not know how to discriminate in the selection of a dentist. We sometimes feel that we have too many of the so-called advertising dentists in this country, but it is a fact that no one of so limited skill would be allowed to perform so difficult an operation as that referred to in any advertising office here. The operators in all our advertising offices are registered men and nine-tenths of them are college graduates. Our situation, it would seem, is much preferrable to that in England on this basis.

Another curious thing about this inquest is that no reputable dentist was called to testify, and the medical man's testimony was sufficient to satisfy the solicitor and the jury that the dentist was only to blame for extracting too many teeth. Any intelligent dentist would have testified that the injection of the gums in the condition described would, in all probability, have been sufficient cause for the gangrenous result. Any one who has studied the re-action of irritant re-agents, such as were indicated by the formula used, would have said at once that cellulitis at least might have been expected from its perenchymatous injection, even in healthy gums. To inject such a preparation into abscessed structures with the force necessary to produce a painless effect would undoubtedly force the pus into the absorbent vessels and blood currents, and lift the periosteum from the bone causing local necrosis and systemic infection. It isn't surprising that a physician might not think this possible, but had an intelligent dentist been called, he would certainly have offered this suggestion to the jury at least. As it is, we suppose this ignoramus will go on taking the lives of innocent people with the consent of those whose business it should be to mercifully care for the innocent. The time will come when the extraction of teeth will be looked upon as a true surgical operation, which it is, and the sooner the general public is educated out of the idea that it can be done by the barber or any other "jack of all trades" for twenty-five cents, the better it will be for the people. Similar fatalities may occur from the extraction of a single tooth when the conditions are right for systemic infection. Of course the extent of the wound in this case bears out the physician's diagnosis in part, as it helped to accentuate the infection because of the prolonged period of healing.

**The National Meetings.**

The next important dental meetings to be held are those of the combined national societies which meet at Atlanta, Ga., beginning with the National Faculties and Examiners Associations, September 14th, and the National Dental Associations, September 18th to 21st. These organizations with several other minor organizations, mark an important event in our professional life, as their actions are often of the greatest consequence to the profession. For a long time the great struggles have taken place in the arena of the National Association of Dental Faculties, but since the National Association of Dental Examiners has taken a notion of setting standards, the scene of most interest has shifted to that body. There are indications, also, that a political clash of no small significance is to take place in the meetings of the National Dental Association this year. It is unfortunate that the meetings are to be held so far from the centers of professional interest, and also at a time when many of the most conservative supporters of these organizations cannot be present. It is hoped that all who can will attend and that moderation and the best interests of the profession may prevail in all the deliberations.

